# Supplementary material for: Stromal Hedgehog Signaling Is Associated with Favorable Outcomes in Pancreatic Cancer
Source: Int J Mol Sci. 2025 May 28;26(11):5200. doi: 10.3390/ijms26115200 (PMC12154493; doi:10.3390/ijms26115200)
Supplement: Supplementary file 1 [file ijms-26-05200-s001.zip › Figure S1 and Table S1 caption.pdf]

Figure S1: **Expression of Hh signaling elements in PDAC cellular compartments**. (**a**) A species-segregated PDX RNA-seq dataset (E-MTAB-683034; [23]) was utilized to compare the expression of Hh ligands and downstream signaling elements between tumor cells and the stroma. Data are presented as a heatmap presenting log2 Zscore values of annotated transcripts. (**b**) The same dataset was utilized to correlate epithelial Hh ligands (Zscore) to stromal signatures. Moffit normal and activated stroma signatures[30] as well as Elyada iCAF and myCAF signatures were utilized[3]. Mouse reads represent the stroma, human reads are tumor-derived. Correlation coefficients and significance were calculated and included on the graphs.

Table S1: **Identified gene signatures in this work**.
