# Supplementary figures and images for: Stromal Hedgehog Signaling Is Associated with Favorable Outcomes in Pancreatic Cancer
Source: Int J Mol Sci. 2025 May 28;26(11):5200. doi: 10.3390/ijms26115200 (PMC12154493; doi:10.3390/ijms26115200)

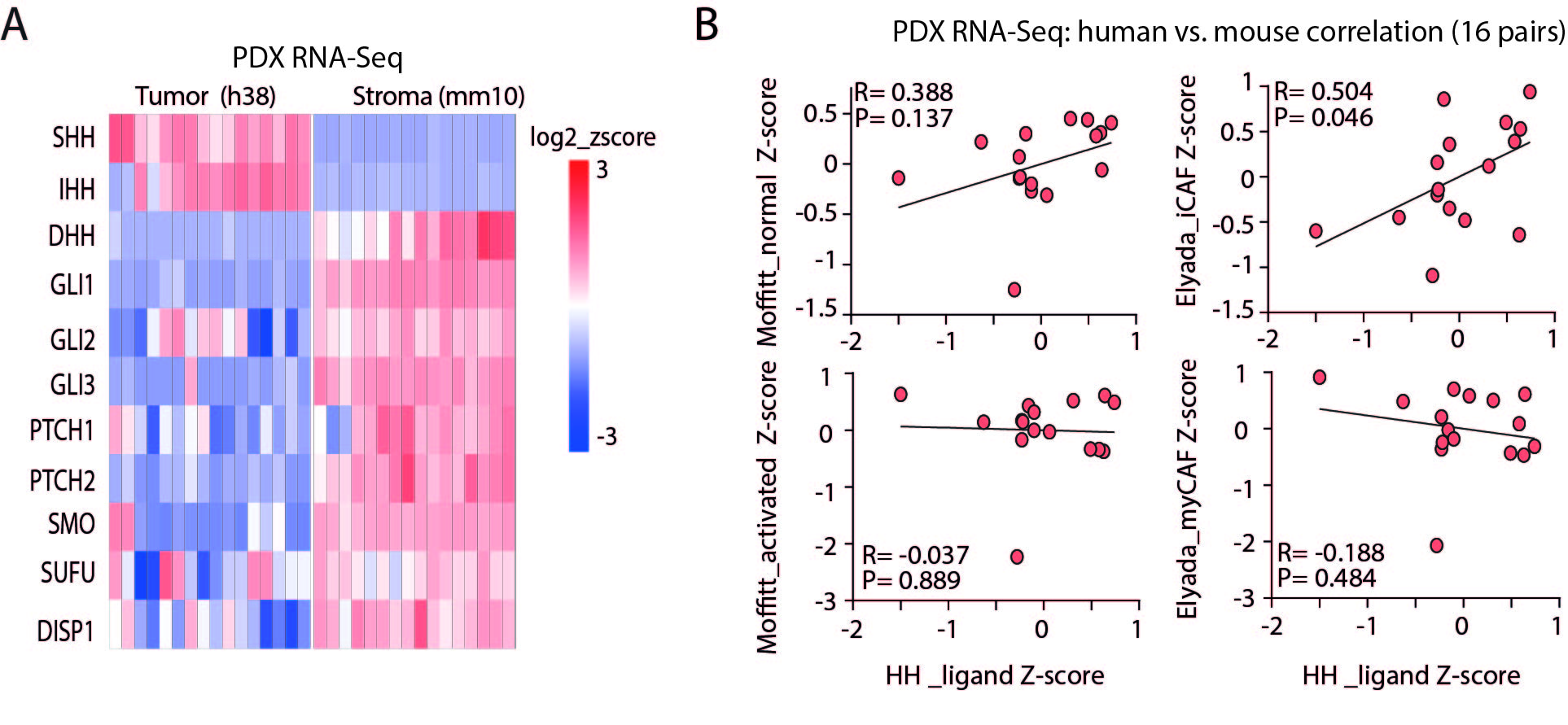

Supplement: Supplementary file 1 [file ijms-26-05200-s001.zip › Figure S1.jpg]
